# VitalDB Arrhythmia Database: An Anesthesiologist-Validated Large-scale Intraoperative Arrhythmia Dataset with Beat and Rhythm Labels

**DOI:** 10.1038/s41597-026-07076-8

**Published:** 2026-03-20

**Authors:** Da-In Eun, Kayoung Shim, Hyunsoo Lee, Yeji Lim, Hanbyeol Lim, Hyeonhoon Lee, Jiwon Lee, Hyung-Chul Lee

**Affiliations:** 1 Interdisciplinary Program in Medical Informatics, Seoul National University, Seoul, Republic of Korea; 2 Healthcare AI Research Institute, Seoul National University Hospital, Seoul, Republic of Korea; 3 Department of Anesthesiology and Pain Medicine, Seoul National University College of Medicine, Seoul National University Hospital, Seoul, Republic of Korea; 4 Department of Anesthesiology and Pain Medicine, Anesthesia and Pain Research Institute, Yonsei University College of Medicine, Seoul, Republic of Korea; 5 Department of Transdisciplinary Medicine, Seoul National University Hospital, Seoul, Republic of Korea; 6 Department of Medicine, Seoul National University College of Medicine, Seoul, Republic of Korea

## Abstract

Intraoperative cardiac arrhythmias present distinct characteristics compared to non-surgical environments, yet publicly available electrocardiogram (ECG) databases have primarily focused on ambulatory or intensive care environments. To address this gap, we present the VitalDB Arrhythmia Database, a comprehensive collection of intraoperative ECG recordings with beat and rhythm labels specifically designed for developing and validating arrhythmia detection algorithms in surgical patients. The database comprises 734,528 seconds of continuous ECG data from 482 surgical patients, with a median annotated recording duration of 20 minutes. It contains over 660,000 annotated heartbeats across four beat types and 10 distinct rhythm categories. To efficiently process the extensive source data, we developed a custom deep learning beat classifier that serves as an automated screening tool for arrhythmia candidate segments. All annotations underwent rigorous validation by five anesthesiologists, with each segment independently reviewed by at least two anesthesiologists, and 9.3% required full committee consensus. Inter-rater reliability analysis demonstrated excellent agreement with an overall Cohen’s kappa of 0.930 ± 0.130. This publicly accessible resource provides the research community with clinically validated intraoperative arrhythmia data, facilitating the development of robust arrhythmia detection algorithms and enabling multimodal analysis to investigate the hemodynamic impact of intraoperative arrhythmias.

## Background & Summary

Multiple factors contribute to arrhythmia during surgery, including airway manipulation, hypovolemia, hypoxemia, electrolyte imbalances, metabolic disturbances, anesthetic agents, and temperature fluctuations^1,2^. The incidence of intraoperative arrhythmias varies significantly based on surgery type and patient comorbidities, occurring in approximately 50–70% of patients undergoing general anesthesia for various surgical procedures^1,3^. While most perioperative arrhythmias are benign and transient, they can require immediate intervention^4,5^. Rapid recognition and timely management of life-threatening arrhythmias are crucial, as delayed or inappropriate treatment can lead to hemodynamic instability, prolonged hospital stays, and increased morbidity and mortality^1,5^.

Given the clinical significance of perioperative arrhythmias and the imperative for timely detection, the availability of expert-labeled intraoperative datasets is essential. While artificial intelligence (AI)-driven electrocardiogram (ECG) classifiers have achieved high accuracy using existing databases, these resources predominantly consist of 12-lead ECGs or ambulatory Holter recordings^6–9^. Consequently, their clinical utility in an intraoperative setting remains limited due to the scarcity of labeled data that capture unique intraoperative challenges, such as specific artifacts and rapid physiological shifts.

To address this critical gap, we introduce the VitalDB Arrhythmia Database, a publicly accessible resource derived exclusively from intraoperative patient monitoring, featuring beat and rhythm annotations rigorously reviewed by experienced anesthesiologists^10^. The scarcity of such datasets has largely stemmed from the need for expert manual review to interpret complex, perioperative-specific rhythm changes. By overcoming these challenges through a systematic clinical validation process, we have curated a comprehensive resource that not only facilitates the development of detection algorithms robust to real-world clinical settings but also enables the evaluation of model performance within the unique context of the surgical environment. Additionally, we release the ECG beat classifier algorithm as open-source software to promote transparency and reproducible research.

The primary purpose of releasing this database, together with open-source algorithmic tools, is to accelerate the integration of technical innovation into clinical practice in perioperative care. Relative to existing datasets that predominantly feature ambulatory recordings, our specific contribution is providing a large-scale, high-fidelity repository of intraoperative ECG data—capturing unique surgical artifacts and rapid physiological shifts—complete with clinically validated beat and rhythm labels. Intended use cases may include developing and validating deep learning models for single-lead ECG waveforms and clinical decision support systems that correlate rhythm disturbances with other hemodynamic changes in perioperative settings.

## Methods

This study is based on our previous work, the VitalDB open dataset hosted on Physionet^6,11,12^, a large-scale, publicly accessible resource containing high-resolution, multi-parameter biosignals from 6,388 non-cardiac surgical cases collected at Seoul National University Hospital^12^. The data was captured prospectively using the Vital Recorder software system, which records time-synchronized data from various anesthetic and patient monitoring devices, including continuous ECG waveforms recorded at a sampling rate of 500 Hz with 16-bit resolution.

### Ethics statement

The collection of the VitalDB open dataset has been approved by the institutional review board of Seoul National University Hospital (H-1408-101-605), and the construction of the data repository has also been registered at a publicly accessible clinical trial registration site (ClinicalTrial.gov, NCT02914444). The IRB exempted our current study from review due to the use of a deidentified public dataset.

### De-identification

As this dataset is derived from the VitalDB open dataset, we strictly adhere to the de-identification protocols established by the parent database^12^. To ensure patient privacy and compliance with the HIPAA Safe Harbor standard, all direct identifiers were removed and replaced with randomized case IDs. Additionally, temporal anonymity was preserved by converting absolute dates into relative timestamps; the start of each recording was standardized to time zero (t = 0), with all subsequent events expressed as the number of seconds elapsed from this reference point.

### ECG beat classifier development

To efficiently screen the arrhythmia candidate segments from the VitalDB open dataset, we developed UniMS-ECGNet, a deep learning algorithm for ECG beat classification and R-peak detection. The architecture employs multiple kernel sizes (50 ms, 150 ms, 310 ms) that correspond to clinically relevant temporal windows optimized to capture diverse morphological features, from rapid QRS transitions to broader P and T wave patterns and conduction abnormalities. The algorithm was trained on data from the VitalDB open dataset, MIT-BIH Arrhythmia Database^6,7^, and CU Ventricular Tachyarrhythmia Database^6,13^.

From the VitalDB open dataset, representative ECG segments from 1,956 surgical patients were selected and manually annotated by an anesthesiologist to provide training data for this classifier. R-peak positions and beat labels for VitalDB and CU Ventricular Tachyarrhythmia Database were manually annotated by an anesthesiologist for this classifier. The model performs five-class classification: normal beats, supraventricular beats, ventricular beats, unclassifiable beats, and false peak detections. The model achieved an overall accuracy of 97.08% on the MIT-BIH Arrhythmia Database.

### Automated arrhythmia candidate selection

We screened the entire VitalDB open dataset using UniMS-ECGNet to find potential arrhythmia candidate segments based on the following criteria: 1) Sequence of three or more consecutive abnormal beats. 2) Segments with high R-R interval variability (coefficient of variation > 0.1) to capture irregular rhythms. 3) Segments with patterned ectopy (e.g., bigeminal pattern) of either atrial or ventricular origin. These screening criteria were established based on clinical rationale, as ventricular tachycardia is defined as three or more consecutive ventricular beats^14^, and irregular arrhythmias such as atrial fibrillation typically exhibit a coefficient of variation exceeding 0.1^15^.

All screened segments underwent manual review. Segments exhibiting noise artifacts that resulted in artificially elevated coefficient of variation values or false beat detections were excluded from the labeling dataset. To balance the review burden associated with high-sensitivity screening while maximizing dataset diversity, we annotated one representative segment per case from the selected arrhythmia candidates. Representative examples corresponding to these screening criteria are illustrated in Figure S1. The distribution of coefficient of variation values across all final labeled segments is presented in Figure S2. Furthermore, to evaluate the impact of varying the CV cutoff on dataset completeness, we performed a sensitivity analysis.

#### Beat annotation

Individual ECG beats were classified into one of four primary classes, as summarized in Table 1. The beat classification scheme was primarily adapted from the Association for the Advancement of Medical Instrumentation EC57 standard^16^. Based on this framework and given that atrial fibrillation is a supraventricular arrhythmia^14^, we classified all beats occurring during atrial fibrillation as supraventricular beats to distinguish them from beats of sinus node origin. Examples of ambiguous cases, where discerning the sinus node of origin was challenging, are provided in Figure S3.

**Table 1:** Beat label criteria.

Beat Label	Individual ECG beats
Normal beat (N)	Left bundle branch block
	Normal ECG beat
	Right bundle branch block
Supraventricular beat (S)	Atrial premature beats
	Abnormal atrial premature beats
	Atrial escape beat
	Borderline escape beat
	Borderline premature beat
	Supraventricular premature beat
	Beats during atrial fibrillation
Ventricular beat (V)	Ventricular premature beat
	Ventricular fusion heartbeat
	Ventricular escape beat
Unclassifiable beat (U)	Beats with a narrow QRS complex, but P wave morphology or T wave morphology cannot be evaluated due to noise

#### Rhythm annotation

Each continuous segment was assigned one of ten final rhythm labels: 1) Atrial Fibrillation, 2) Atrioventricular Block, 3) Normal Sinus Rhythm, 4) Sinus Node Dysfunction, 5) Patterned Atrial Ectopy, 6) Patterned Ventricular Ectopy, 7) Supraventricular Tachyarrhythmia, 8) Ventricular Tachyarrhythmia, 9) Wandering Atrial Pacemaker/Multifocal Atrial Tachycardia, and 10) Unclassifiable.

##### Signal quality assessment

**Fig. 1:**
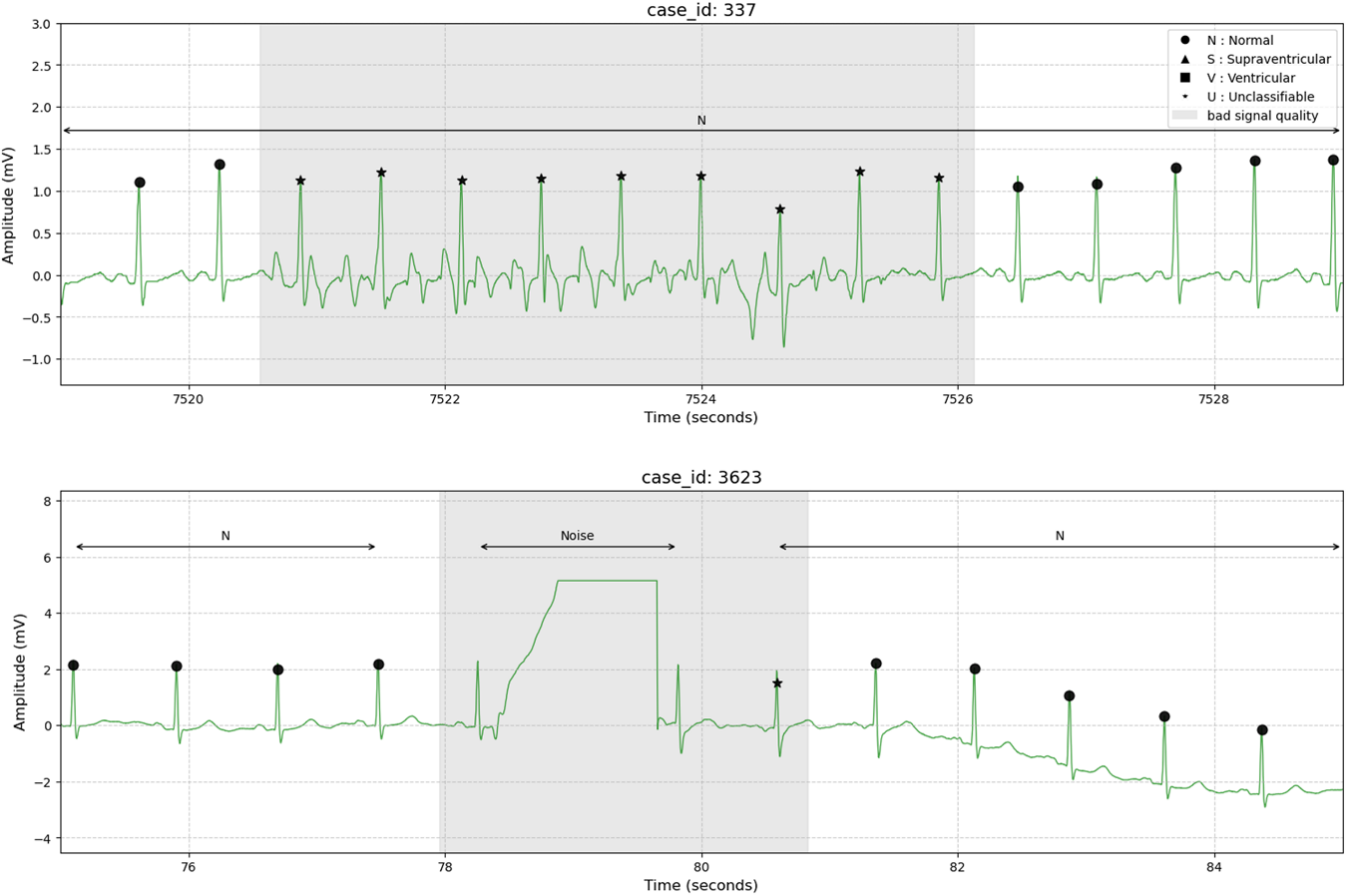
Examples of ECG signal quality labeling. Individual beat labels are marked at each beat position. Rhythm labels are indicated above arrows that denote the corresponding segments. Segments with bad signal quality are highlighted with gray shading. The upper panel shows an example of a normal sinus rhythm (N; Normal Sinus Rhythm) with a bad signal quality segment. The lower panel demonstrates a case where a normal sinus rhythm and noise segments coexist, with bad signal quality present.

A ‘bad signal quality’ flag was used for segments where QRS complexes were visible, but their morphology was obscured. This flag could be applied concurrently with another rhythm label, as the underlying rhythm was still interpretable. In contrast, the ‘Noise’ label was applied for segments where severe artifacts rendered QRS complexes undetectable. Consequently, these segments do not contain any beat-level annotations. Example cases were presented in Fig. 1 and Figure S4.

##### Transient conduction block events

**Fig. 2:**
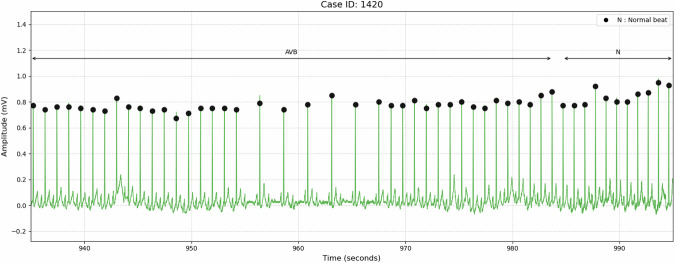
Example of transient atrioventricular block. Individual beat labels are marked at each beat position. Rhythm labels are indicated above arrows that denote the corresponding segments (AVB; Atrioventricular Block). The figure demonstrates a transient atrioventricular block event where a 40-second window (20 seconds before and after the block occurrence) is labeled with the same atrioventricular block rhythm classification to provide clinical context.

For non-sustained conduction blocks (transient AV block or sinoatrial block), a 40-second window (20 seconds before and after the event) was labeled with the same rhythm classification to provide clinical context (Fig. 2).

##### Patterned arrhythmias

Arrhythmias with patterns like bigeminy, trigeminy, and quadrigeminy were grouped into single categories (Patterned Atrial or Ventricular Ectopy). These patterns were considered to have ended when five or more consecutive normal beats occurred.

##### Sinus node dysfunction

In accordance with the 2018 American College of Cardiology/American Heart Association/Heart Rhythm Society (ACC/AHA/HRS) Guideline^17^, this category encompasses rhythms resulting from sinus node failure or the failure of subsidiary pacemakers, including sinus arrhythmia, sinoatrial block, and junctional rhythm.

##### Unclassifiable

**Fig. 3:**
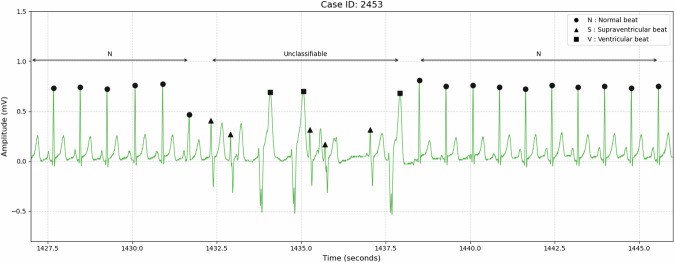
Example of Unclassifiable rhythm in the rhythm transitional segment. Individual beat labels are marked at each beat position. Rhythm labels are indicated above arrows that denote the corresponding segments (N; Normal Sinus Rhythm).

Rhythmic transition zones that could not be assigned a single definitive label were marked as Unclassifiable (Fig. 3).

### Physician review and validation

All automated selections and initial annotations underwent rigorous clinical validation by a panel of five anesthesiologists. Prior to the annotation process, the reviewers collectively reviewed common intraoperative arrhythmia cases and standard guideline documents to establish uniform diagnostic criteria. The review was conducted using a custom-developed labeling tool specifically designed for this study (Figure S5). Each candidate segment was independently labeled by at least two physicians, and we subsequently held regular consensus meetings with the full committee to resolve any disagreements and reach a final decision on each case.

This process resulted in a final dataset from 482 patients, comprising a total of 661,894 validated heartbeats over 734,528 seconds of annotated recordings. The median annotated recording duration was 1,198 seconds (IQR: 1,078–1,199 seconds). The detailed statistics for each rhythm category are presented in Table 2.

**Table 2:** Summary statistics of Annotated Labels.

Rhythm Label	Number of cases	Number of beats	Duration in Seconds
Normal Sinus Rhythm	370	408,420	384,407
Noise	250	—	67,734
Atrial fibrillation	111	163,270	121,888
Patterned Ventricular Ectopy	109	24,069	47,481
Supraventricular Tachyarrhythmia	109	6,416	14,799
Ventricular Tachyarrhythmia	88	1,598	9,927
Patterned Atrial Ectopy	85	20,326	40,600
Sinus Node Dysfunction	66	23,141	31,942
Wandering Atrial Pacemaker/Multifocal Atrial Rhythm	26	10,132	9,630
Atrioventricular Block	10	4,323	5,486
Unclassifiable	6	199	631

## Data Records

The VitalDB Arrhythmia Database, comprising all beat and rhythm annotations generated in this study, is available at PhysioNet (10.13026/axd6-wm13)^18^ and the GitHub repository (https://github.com/vitaldb/arrdb)^10^. It is composed of a general metadata file and an annotation folder that contains 482 annotation files in CSV format.

### General metadata

We provide general metadata about the records in a single CSV file, metadata.csv. This file includes comprehensive patient demographics and surgical parameters from the original VitalDB dataset, along with summary statistics derived from the annotated ECG data. The file contains the following key columns related to the annotations:

#### case_id

The unique identifier for each case, which corresponds to the original VitalDB file name and is used in the annotation file naming (Annotation_file_[case_id].csv).

#### analysis_start_time_sec

The timestamp in seconds of the first annotated beat in the recording.

#### analysis_end_time_sec

The timestamp in seconds of the last annotated beat in the recording.

#### analyzed_duration_sec

The total duration in seconds of the analyzed and labeled portion of the recording.

#### total_beats

The total number of detected heartbeats in the annotation file.

#### rhythm_classes

A list of all unique rhythm labels present in the annotation file.

### Annotation files

Each case has a corresponding CSV file with detailed beat and rhythm annotations. The annotation files are provided in the format “Annotation_file_[case_id].csv” for each case.

#### time_second

The timestamp of the R-peak in seconds, measured from the beginning of the recording.

#### beat_type

The classification of the individual heartbeat.

#### rhythm_label

The overall heart rhythm label for the segment in which the beat occurs.

#### bad_signal_quality

A boolean marker (True/False) indicating if the beat is located within a segment of excessive noise or poor signal quality.

#### bad_signal_quality_label

A label indicating the start or end of a bad signal quality segment (e.g., ‘Start1’, ‘End1’). This column is empty for rows not marking these specific boundaries.

### ECG Waveform Data

The original, unprocessed waveform data for each case can be accessed and downloaded directly from PhysioNet (10.13026/czw8-9p62)^11^.

## Technical Validation

### Clinical validation

Arrhythmia candidate segments were initially identified through automated selection, and a sensitivity analysis evaluating the applied CV cutoffs is detailed in Table S1. Of the 482 cases in the database, 45 cases (9.3%) required a final consensus decision by the full committee to resolve initial disagreements between the annotators.

Inter-rater agreement was assessed by calculating Cohen’s kappa coefficients between initial annotations and final consensus labels. The overall Cohen’s kappa was 0.930 ± 0.130, indicating excellent inter-rater agreement. Beat-specific kappa values were 0.942 ± 0.185 for normal beats (N), 0.916 ± 0.199 for unclassifiable beats (U), 0.942 ± 0.220 for ventricular beats (V), and 0.899 ± 0.243 for supraventricular beats (S). The beat-level confusion matrix is presented in Table S2, and Cohen’s kappa coefficients stratified by rhythm class are provided in Table S3. Our analysis showed robust agreement for ventricular arrhythmias, whereas comparatively lower agreement was observed for supraventricular rhythms, reflecting the inherent clinical challenge of discerning subtle atrial activity in surgical environments.

### Technical validation

To assess the utility of our dataset, we developed a binary classification model to distinguish between normal and abnormal rhythms using a one-dimensional convolutional neural network.

For model training, a 5-second ECG segment was labeled as ‘normal’ only if it was part of a continuous 45-second window entirely annotated as normal sinus rhythm to exclude rhythm transition zones from the ‘normal’ class. Conversely, any segment with a rhythm label other than ‘normal sinus rhythm’ was classified as ‘abnormal’. To ensure a clear assessment during evaluation, segments of normal sinus rhythm that included premature beats were omitted.

The model was trained exclusively using the VitalDB Arrhythmia Database, which was partitioned at the patient level into an 8:2 training and validation split to ensure no patient appeared in both sets. The model’s performance was then evaluated on the validation set from the VitalDB Arrhythmia Database and cross-validated on the entire MIT-BIH Arrhythmia Database.

**Fig. 4:**
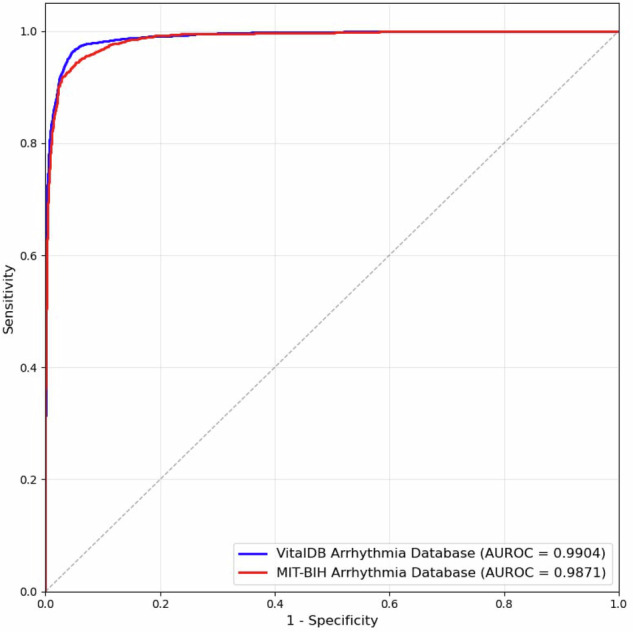
Receiver Operating Characteristic (ROC) curves of the arrhythmia detection model.

The performance of the binary classification model is detailed in Table 3. The model achieved an accuracy of 0.928, a sensitivity of 0.848, a specificity of 0.988, and an F1-score of 0.910 on the held-out validation set from our database. When cross-validated on the external MIT-BIH Arrhythmia Database, the model achieved an accuracy of 0.949, a sensitivity of 0.928, and an F1-score of 0.925. The Area Under the Receiver Operating Characteristic (AUROC) was 0.9904 for the VitalDB Arrhythmia Database and 0.9871 for the MIT-BIH Arrhythmia Database (Fig. 4).

**Table 3:** Performance metric of the arrhythmia detection model on the VitalDB Arrhythmia Database and MIT-BIH Arrhythmia Database.

	VitalDB Arrhythmia Database	MIT-BIH Arrhythmia Database
Accuracy	0.928	0.949
Sensitivity	0.848	0.928
Specificity	0.988	0.959
PPV	0.981	0.922
NPV	0.898	0.963
F1-Score	0.910	0.925

PPV, positive predictive value; NPV, negative predictive value.

## Usage Notes

A step-by-step guide for merging and utilizing the VitalDB Arrhythmia Database^18^ alongside the VitalDB open dataset^11^ is provided in the UsageNote.ipynb Jupyter Notebook, available in our GitHub repository^10^.

### Download annotations

Download annotation files from PhysioNet (10.13026/axd6-wm13)^18^ or the GitHub repository (https://github.com/vitaldb/arrdb)^10^.

### Install required packages

Install the Python packages to access waveform data and handle annotations (pip install vitaldb pandas numpy matplotlib).

### Load ECG waveforms and annotations

Use the case_id to download the ECG waveform from VitalDB and load the corresponding annotation file.

import vitaldb

import pandas as pd

import numpy as np

# Example case_id

case_id = 337

#Load ECG waveform from VitalDB (sampled at 500 Hz)

vals = vitaldb.load_case(case_id, [‘SNUADC/ECG_II’], 1/500)

ecg_data = vals[‘SNUADC/ECG_II’]

# Load annotation file

annotation_file = f’Annotation_file_{case_id}.csv’

annotations = pd.read_csv(annotation_file)

# Display the structure of annotations

print(annotations.head())

# Extract specific columns

time_seconds = annotations[‘time_second’].values

beat_types = annotations[‘beat_type’].values

rhythm_labels = annotations[‘rhythm_label’].values

signal_quality = annotations[‘bad_signal_quality’].values

## Supplementary information

Supplementary Information

## Data Availability

The VitalDB Arrhythmia Database, including all beat and rhythm annotations generated and analyzed during the current study, is publicly available at PhysioNet (10.13026/axd6-wm13)^18^ and at the GitHub repository (https://github.com/vitaldb/arrdb)^10^. The dataset is made publicly available under the Creative Commons Attribution 4.0 International (CC BY 4.0) License. This license places no restrictions on commercial or derivative use, fostering open collaboration and the development of third-party algorithms, provided the original work is properly cited.

## Code availability

The screening algorithm used to create this database is publicly available as an open-source tool through the Pyvital Python package. Detailed usage instructions and implementation examples can be found in the Vital_beat_noise_detection.ipynb notebook at https://github.com/vitaldb/arrdb10.
