# Supplementary material for: VitalDB Arrhythmia Database: An Anesthesiologist-Validated Large-scale Intraoperative Arrhythmia Dataset with Beat and Rhythm Labels
Source: Sci Data. 2026 Mar 20;13:838. doi: 10.1038/s41597-026-07076-8 (PMC13237132; doi:10.1038/s41597-026-07076-8)
Supplement: Supplementary file 1 — Supplementary Information [file 41597_2026_7076_MOESM1_ESM.docx]

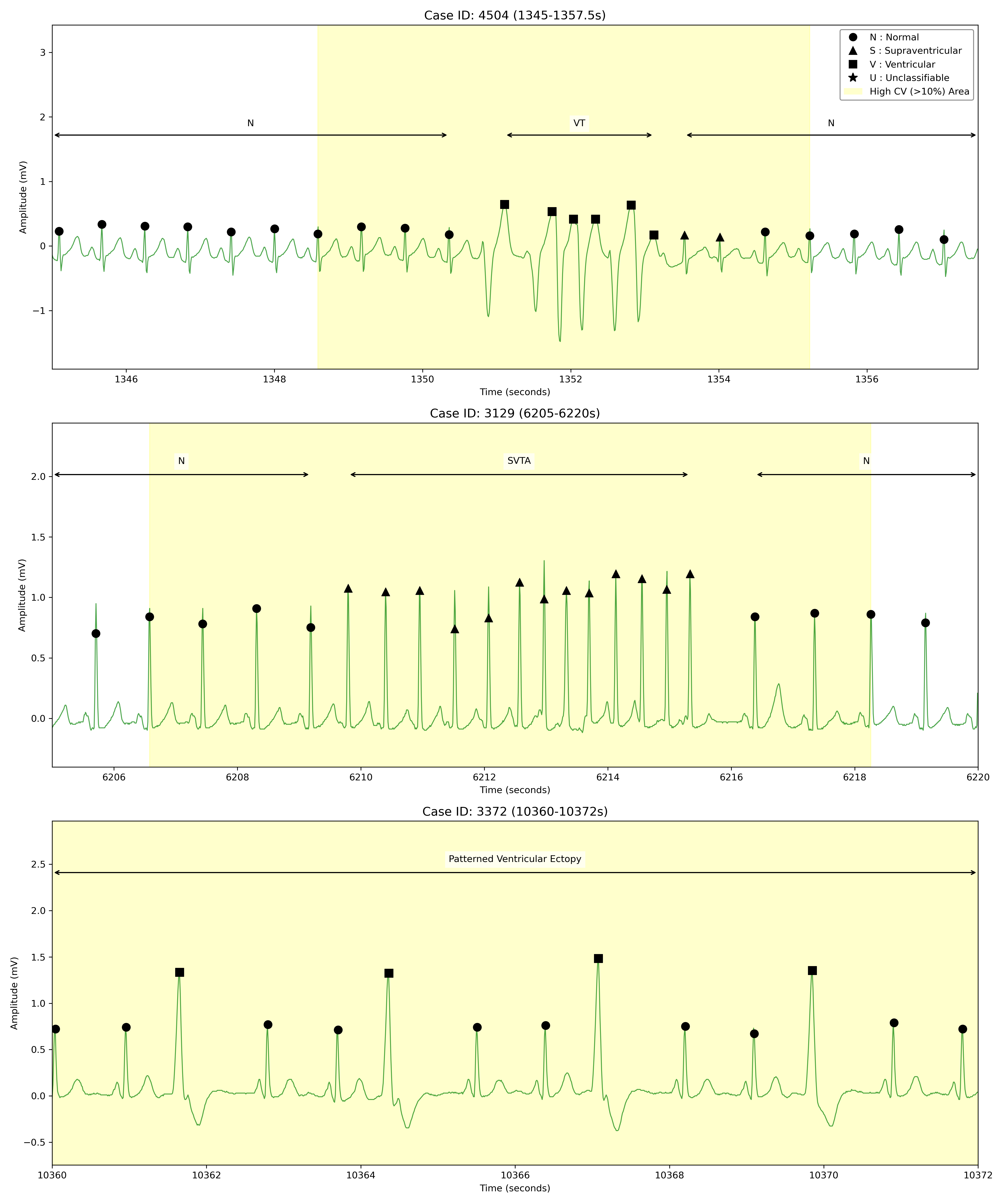


**Figure S1. Examples of arrhythmia candidate selection.** Individual beat labels are annotated at each beat position, and rhythm labels are indicated above arrows denoting the corresponding segments. Regions with a coefficient of variation (CV) exceeding 0.1 are highlighted with yellow shading. The top panel displays a sequence of three or more consecutive ventricular (V) beats; the middle panel shows three or more consecutive supraventricular (S) beats; and the bottom panel depicts a case exhibiting a consecutive rhythmic pattern. N=Normal Sinus Rhythm; VT=Ventricular Tachycardia; SVTA=Supraventricular Tachyarrhythmia.


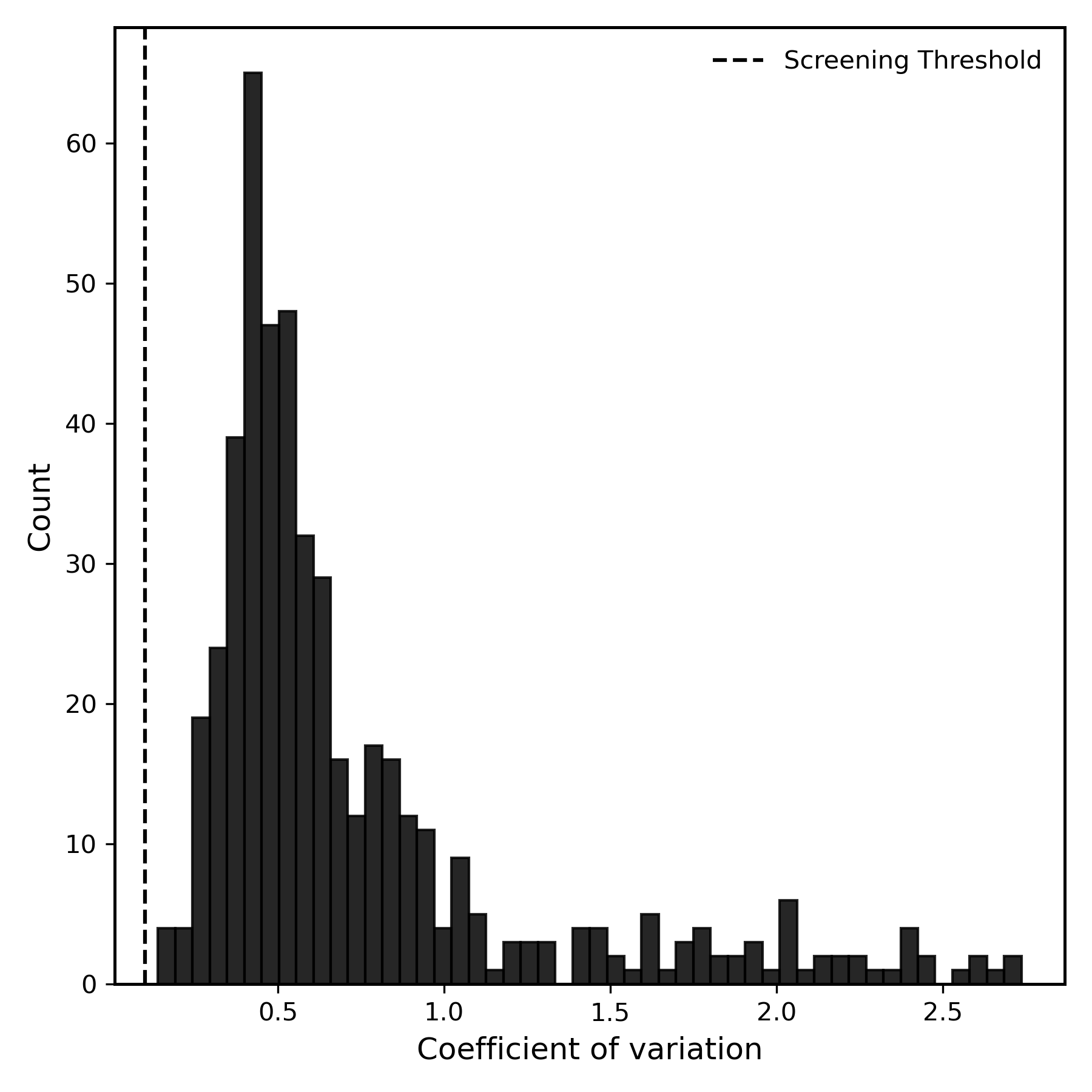


**Figure S2. Distribution of R-R interval Coefficient of Variation (CV) across annotated arrhythmia segments.** The histogram illustrates the frequency of CV values in the final labeled dataset. The vertical dashed line indicates the screening threshold (CV = 0.1) used for candidate selection.


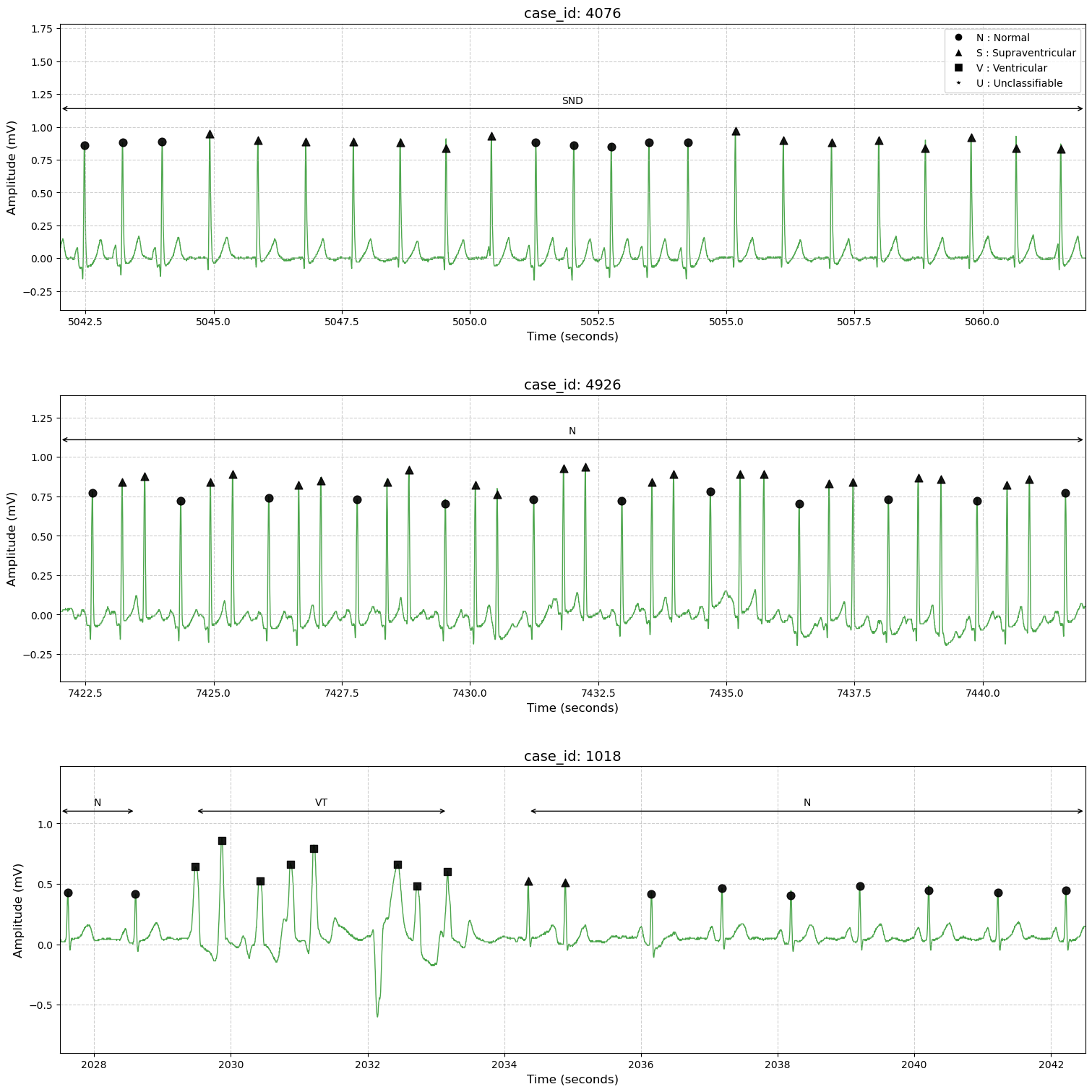
**Figure S3. Examples of adjudication criteria for ambiguous beats.**

Individual beat labels are annotated at each beat position. The top panel displays a beat classified as a supraventricular beat due to a distinct P wave morphology and sudden rate change compared to the baseline normal beats; the middle panel shows a case with multiple ectopic atrial sources where normal beats were identified based on consistent P wave morphology; and the bottom panel depicts a beat immediately following ventricular tachycardia that was classified as a supraventricular beat based on morphological comparison with the subsequent normal sinus rhythm. N=Normal Sinus Rhythm; SND=Sinus Node Dysfunction; VT=Ventricular Tachycardia.


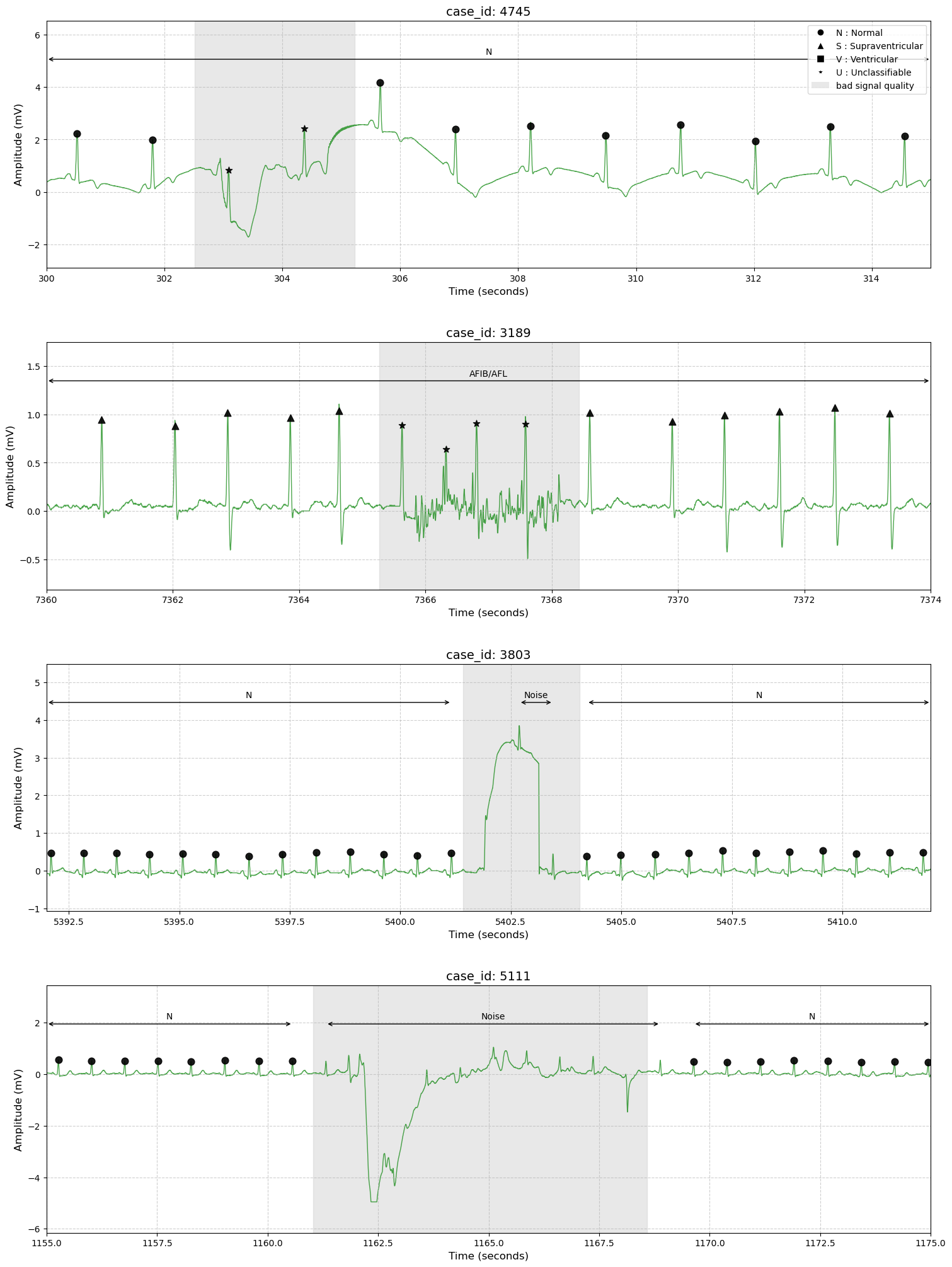


**Figure S4. Examples of ECG signal quality labeling.**

Individual beat labels are marked at each beat position. Rhythm labels are indicated above arrows that denote the corresponding segments. Segments with bad signal quality are highlighted with gray shading. N=Normal Sinus Rhythm; AFIB/AF=Atrial Fibrillation/Atrial Flutter.


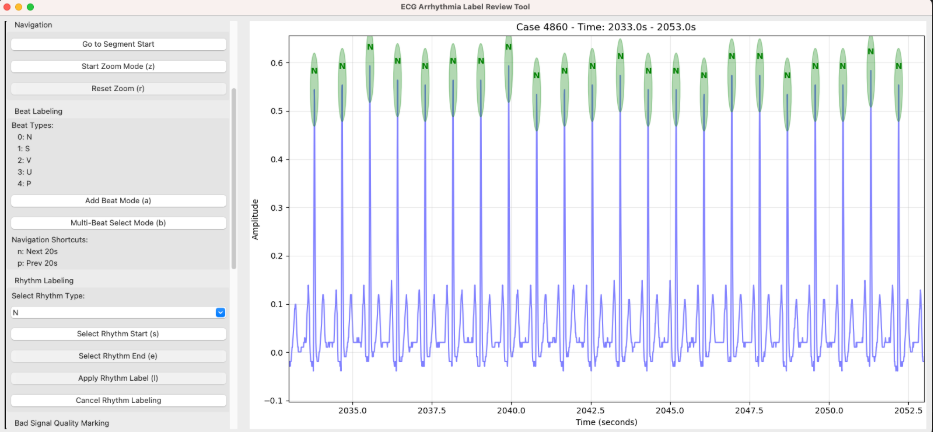
**Figure S5. The custom-developed ECG labeling tool for physician annotation.** This tool enables efficient manual review by displaying the ECG waveform with interactive controls. Key features include R-peak adjustment, beat-type modification, annotation of noise and poor-signal-quality segments, and rhythm category assignment. N=Normal; S=Supraventricular; V=Ventricular; U=Unclassifiable.

**Table S1.** Case-level sensitivity analysis of automated screening across different coefficient of variation (CV) thresholds.

| CV threshold (%) | Total Arrhythmia Candidates | True Arrhythmia Cases |
| --- | --- | --- |
| > 10.0 | 3,896 | 193 |
| > 15.0 | 3,894 | 192 |
| > 20.0 | 3,883 | 188 |
| > 25.0 | 861 | 95 |

**Table S2.** Beat-level confusion matrix between initial annotations and final annotations. N=Normal; S=Supraventricular; V=Ventricular; U=Unclassifiable.

|  | Initial Annotations | | | | |
| --- | --- | --- | --- | --- | --- |
|  |  | **N** | **S** | **V** | **U** |
| Final Annotations | **N** | 0.989 | 0.008 | 0.000 | 0.002 |
|  | **S** | 0.006 | 0.979 | 0.012 | 0.003 |
|  | **V** | 0.000 | 0.000 | 0.999 | 0.001 |
|  | **U** | 0.002 | 0.002 | 0.001 | 0.995 |

**Table S3.** Cohen’s kappa stratified by rhythm class

| Rhythm Label | Cohen's Kappa |
| --- | --- |
| Patterned Ventricular Ectopy | 0.990 |
| Unclassifiable | 0.981 |
| Wandering Atrial Pacemaker / Multifocal Atrial Tachycardia | 0.961 |
| Ventricular Tachycardia | 0.936 |
| Sinus Node Dysfunction | 0.917 |
| Normal Sinus Rhythm | 0.915 |
| Patterned Atrial Ectopy | 0.906 |
| Atrial Fibrillation / Flutter | 0.818 |
| Supraventricular Tachyarrhythmia | 0.645 |
| Atrioventricular Block | 0.596 |
